# Glutathione Preservation during Storage of Rat Lenses in Optisol-GS and Castor Oil

**DOI:** 10.1371/journal.pone.0079620

**Published:** 2013-11-19

**Authors:** Thomas Holm, Martin Rocho Brøgger-Jensen, Leif Johnson, Line Kessel

**Affiliations:** Department of Ophtalmology, Glostrup Hospital, University of Copenhagen, Copenhagen, Denmark; Case Western Reserve University, United States of America

## Abstract

**Background:**

Glutathione concentration in the lens decreases in aging and cataractous lenses, providing a marker for tissue condition. Experimental procedures requiring unfrozen lenses from donor banks rely on transportation in storage medium, affecting lens homeostasis and alterations in glutathione levels. The aim of the study was to examine the effects of Optisol-GS and castor oil on lens condition, determined from their ability to maintain glutathione concentrations.

**Methodology/Principal Findings:**

Rat lenses were stored in the two types of storage media at varying time intervals up to 3 days. Glutathione concentration was afterwards determined in an enzymatic detection assay, specific for both reduced and oxidized forms. Lenses removed immediately after death exhibited a glutathione concentration of 4.70±0.29 mM. *In vitro* stored lenses in Optisol-GS lost glutathione quickly, ending with a concentration of 0.60±0.34 mM after 3 days while castor oil stored lenses exhibited a slower decline and ended at 3 times the concentration. A group of lenses were additionally stored under post mortem conditions within the host for 6 hours before its removal. Total glutathione after 6 hours was similar to that of lenses removed immediately after death, but with altered GSH and GSSG concentrations. Subsequent storage of these lenses in media showed changes similar to those in the first series of experiments, albeit to a lesser degree.

**Conclusions/Significance:**

It was determined that storage in Optisol-GS resulted in a higher loss of glutathione than lenses stored in castor oil. Storage for more than 12 hours reduced glutathione to half its original concentration, and was considered unusable after 24 hours.

## Introduction

The small thiol peptide glutathione (GSH) has a central role in protecting against oxidative stress in the lens and its decreasing concentration and activity with age are believed to be a major factor in the formation of age-related cataract [1]–[3]. Reduced glutathione (GSH) functions alone or as substrate in many enzymatic reactions by serving as an electron donor to highly unstable and reactive molecules [4] and serves to protect thiol groups of membrane proteins and intracellular crystallins from cross-linking by post-translational modifications [5]–[7]. Oxidized glutathione (GSSG) reacts non-enzymatically with protein thiol groups to create protein-GSH (PSSG) mixed disulfides that may function as a reservoir for GSH and serve to protect against disulfide cross-linking of proteins (PSSP) [8]. Increased disulfide-linkages lead to high molecular weight protein aggregation [9], [10], which is directly linked to the loss of transparency in the lens [11], [12].

GSH is replenished by both de novo synthesis in the epithelial layer of the lens in a 2-stage ATP-dependent enzymatic reaction [13], [14] and through the reduction of GSSG by Glutathione Reductase (GSR) [15]. In 80 days old rat lenses, however, glutathione levels dropped in culture by approximately 20% over 24 hours without any significant increase of GSSG [16], suggesting that the lens may lose glutathione by mechanisms unrelated to oxidation.

Given the central role of glutathione in lens homeostasis, measurements of its concentration and oxidation state can be used as an indicator of the redox environment and overall condition of the tissue when used in research. Due to practical conditions, human donor lenses are not usually available for research until several hours post mortem and in the time period between death, isolation of the lens and transport to research facilities in organ culture medium, several factors may have influenced antioxidant activity, degradation and efflux. Freezing of human donor lenses maintains these conditions but will also disrupt the tissue, jeopardizing studies that focus on morphology and physiological/optical functions of whole intact lenses. Storage conditions should ideally minimize such processes and extend the viability of lenses.

The aim of the present study was to examine how the concentrations of oxidized and reduced glutathione change post-mortem in an animal model system in order to obtain information as to whether reliable measurements of glutathione can be obtained using human donor lenses and which conditions best preserve lenses in the *in vivo* state. Rat lenses were stored in different media for various durations of time to mimic the conditions for human donor lens transportation and the state of glutathione under these conditions was determined.

## Materials and Methods

### Ethics Statement

Experiments were approved by the Supervisory Authority on animal Testing of Denmark (dyreforsøgstilsynet: original permit 2009/561-1630, extended permit 2013-15-2934-00804). All animal treatment adhered to the ARVO Statement for the Use of Animals in Ophthalmic and Vision Research, and all efforts were made to minimize suffering of the animals.

### Animals

A total of 86 male albino Sprague-Dawley rats aged 9 weeks (Taconic NTac: SD) were used in these experiments. Rats were killed by carbon dioxide asphyxiation and decapitation.

### Storage media

This study compared the two media: Optisol-GS (Bausch & Lomb 50006-OPT) and castor oil (Sigma-Aldrich 259853). Optisol-GS is a widely used commercial storage media, whereas castor oil is a hydrophobic media consisting mainly of the unsaturated ricinoleic acid as well as a number of saturated fatty acids. An analysis of Optisol-GS medium found a GSSG concentration of 10 μM. This value characterizes a baseline level of glutathione already present in the medium prior to rat lens incubation which would affect accuracy of low glutathione measurements.

### Lens Storage

In the first group of experiments, lenses were removed immediately after death and in the second group of experiments, the eye was left intact within the animal, eyelids taped shut, and the head stored at 4°C for 6 hours. In both sets of experiments, the eyes were partially enucleated and an incision was made just anteriorly of the ora serrata around the circumference of the eye to remove the cornea and iris. Gentle pressure was applied to the sclera and the lens was lifted from the eye cup and freed of vitreous tissue. Lenses were then homogenized immediately or placed in storage media and stored at 4°C for varying time periods of up to 72 hours.

Four to seven lenses were analyzed for each experimental group. The Optisol-GS medium was originally designed for storage of human corneas and since it was found to induce osmotic damage to rat lenses stored for more than 24 hours, 5% BSA (Sigma A4503) was added to reduce the osmotic pressure.

11 week old lenses were stored in Optisol-GS containing 25 mM exogenous GSH, to determine the existence of passive diffusion of glutathione in *in vitro* stored lenses.

### Tissue preparation

After dissection and storage in either Optisol-GS or castor oil, the lenses were washed once in isotonic saline solution (9 g NaCl/L) and placed in lysis buffer consisting of 150 mM NaCl, 50 mM Tris-HCl (pH = 7.4), protease inhibitor (Roche 04 693 124 001) and phosphatase inhibitor (Roche 04 906 837 001). The lenses were mechanically homogenized with an Ultra-Turrax T8 (IKA Labortechnik) and left to lyse for 30 minutes on ice.

Homogenates were centrifuged at 14.000 g at 4°C for 15 minutes, 333 μl of the supernatant was removed and the pellet resuspended in 300 μl lysis buffer. This procedure was conducted three times to maximize extraction and the resulting supernatants (total 1 mL) were pooled for each individual sample. All procedures were performed on ice and samples were stored at −80°C until further analysis could be performed.

### Glutathione measurements

Reduced and oxidized glutathione were measured using a commercially available glutathione luminescence detection kit according to the manufacturer's instructions (Glutathione assay kit, Promega V6912). The kit exhibits a high specificity for reduced glutathione rather than thiols in general. Oxidized glutathione was measured as the difference between the original reading and a reading of total glutathione obtained by adding 0.2 μM of the reducing agent, tris (2-carboxyethyl) phosphine (TCEP; Sigma 646547).

Standard curves were obtained by diluting 0–12.5 μM GSH in lysis buffer and 0–12.5 μM GSH in lysis buffer with 200 uM TCEP. To obtain readings within the standard curve reference, lens samples were diluted 30×, 20× and 10× for samples of lenses 0 to 1 hour after death, 6 hour after death and 24 & 72 hours after death, respectively. All lens samples were analysed in triplicate on a luminescence plate reader (Tecan Infinite M200).

### Glutathione measurement of medium

Measurements performed on Optisol-GS with GSH added in known amounts found only GSSG at all time points analysed, even in samples which were frozen immediately, indicating a high oxidative potential of the Optisol medium.

Measuring glutathione in castor oil was achieved by combining equal amounts of lysis buffer and castor oil and then tumbling these at room temperature for 3 hours. The lysis buffer, now containing glutathione, was subsequently stored at −80°C until analysed.

### High resolution respirometry

Lenses were removed as described above and homogenized in Mir05 medium before being placed in an Oroboros Oxygraph-2 k (Oroboros Instruments, Innsbruck, Austria). Four samples were run simultaneously with a controlled constant temperature of 37°C. Oxygen concentration of the medium and rate of oxygen consumption were monitored and recorded in real-time using DatLab 4.3 software (Oroboros Instruments, Innsbruck, Austria). The samples were allowed to stabilize after which tricarboxylic acid cycle substrates were added (malate (5 mM), pyruvate (5 mM), glutamate (5 mM) and succinate (10 mM) followed by ADP (1 mM). This procedure maximized phosphorylation by the electron transfer system (ETS) by both complex I and II in the coupled state. Finally all electron flow through the ETS was inhibited by the complex III inhibitor antimycin-A (1 μg/ml). The residual oxygen consumption measured was non-mitochondrial. Samples with unstable respiration rates caused the exclusion of measurements from both chambers.

### Data Handling

Raw data obtained from the plate reader, was compared to a standard curve which was run in parallel on the same plate, yielding a concentration result for the 1 μmL lens homogenates. All data series were revised to omit data points deviating more than 80% from the average. This resulted in the exclusion of 2 data points from Optisol-GS 24 hours and 3 data points from Optisol-GS 72 hours.

Calculating the concentration within the actual lenses, we used a standard volume for a rat lens of 42 μL, and gave the following formula:
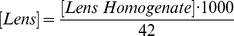



To properly compare glutathione amount in the different volumes of media and lens in the efflux studies, the concentrations were changed to molar amounts using the following formula:
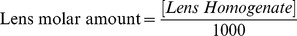


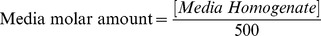



GSSG is comprised of two GSH molecules, although for simplicity and easier comparison GSSG is described as the concentration of single glutathione molecules, resulting in a concentration twice as large as that of actual GSSG.

### Statistical Analysis

The change in parameters across time for each media were analysed with 1-way ANOVA, using Dunnett's post-hoc test to compare each time point with time 0. Parameters in the two media were analysed with 2-way ANOVA and differences at one single time point were compared using the Least Significant Difference (LSD) post-hoc test where p-values were adjusted using the Bonferroni-Holm method.

## Results

### Rat lenses removed immediately after sacrifice of animals

**Figure 1:**
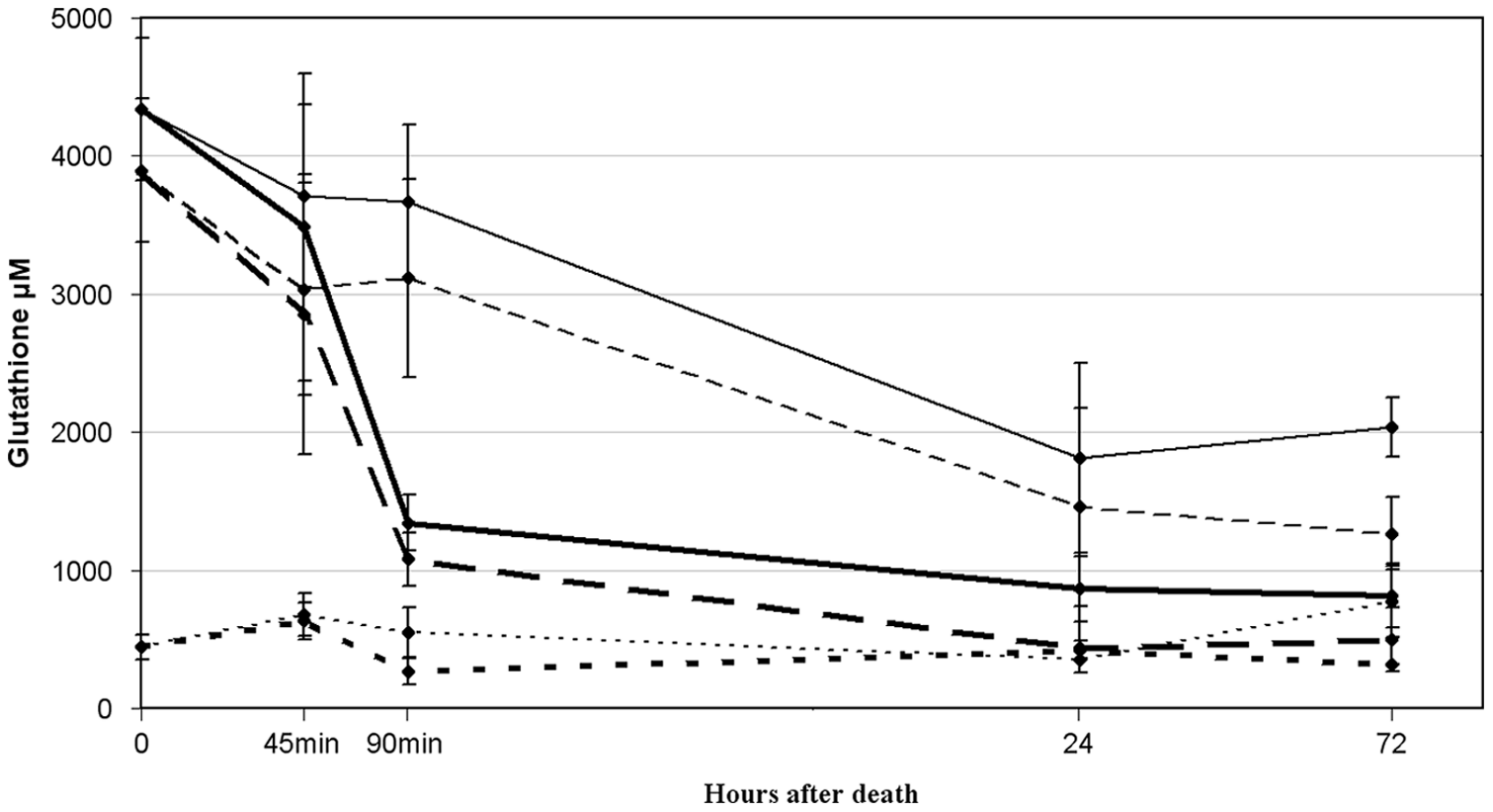
Glutathione of *in vitro* stored lenses. *In vitro* Optisol-GS stored lenses showed a rapid drop in concentration at 1½ hour, which was not observed with castor oil. A significant effect of the storage media is observed after 72 hours, with castor oil stored lenses retaining three times the amount of Optisol-GS stored lenses. Total glutathione is shown as full lines, GSH as large dotted and GSSG as small dotted lines. Bold lines show the progression in Optisol-GS and thin lines in castor oil.

Initially, total glutathione concentration in lenses removed immediately after death was 4.34±0.52 mM, with a GSH value of 3.90±0.52 mM and a GSSG value of 0.44±0.09 mM (Fig 1.). The redox ratio of the post mortem lenses were determined as the GSH/GSSG ratio, which for initial concentrations were calculated to 8.77±2.90.

### Optisol stored lenses

Both total glutathione and GSH showed a rapid drop during the first 1½ hour, followed by a slow decline and reaching a constant level towards 72 hours (Fig 1.). GSSG concentrations saw a small drop during the initial 1½ hour and afterwards balanced itself at the original level. After the initial drop, total glutathione and GSH concentration ended at 1.34±0.20 mM (P<0.05) and 1.08±0.19 mM (P<0.05) respectively. GSSG levels fluctuated during the first 90 min by both increasing 0.63±0.13 mM (P<0.05) and decreasing 0.26±0.09 mM (P<0.05) to statistical significant values before equilibrating back to its original value of approximately 0.4 mM.

The redox ratio of lenses stored in Optisol-GS dropped steadily throughout storage. The drop was affected by the rapid drop of glutathione concentrations during the first 1½ hour to itself drop rapidly to 4.47±1.26 (P<0.05) at 45 min. Afterwards it continued a slow decrease to equilibrium levels of which varied around a ratio of 1. The drop in redox ratio exhibited a statistical significant development (P<0.0001).

Diffusion mechanisms of glutathione were studied by storing lenses in Optisol-GS, supplemented with exogenous GSH. Lenses stored for 2 hours in Optisol-GS +25 μM GSH (n = 10) retained 46% more GSH compared to lenses stored in buffer free of GSH (n = 10) (p<0.001) (data not shown).

### Glutathione recovery in Optisol-GS medium

Recovery of glutathione in the Optisol-GS medium itself increased over time to statistical significant values. Total glutathione recovered in media reached a maximum of 30 nmol, and all glutathione was recovered as GSSG, with only trace amount of GSH (data not shown).

### Castor oil stored lenses

With lenses stored in castor oil, the total glutathione and GSH content declined steadily throughout the 72 hours to 2.04±0.21 mM (P<0.05) and 1.26±0.26 mM (P<0.05) respectively (Fig 1), retaining a generally higher concentration throughout the storage.

GSSG retained a constant value except at 72 hours where the concentration rose to 0.77±0.25 mM (P<0.05).

The redox ratio of castor oil stored lenses dropped similar to Optisol lenses at 45 min to 4.83±2.07 (P<0.05), and afterwards retained a generally higher value only to end at a value equal to that of Optisol-GS. The development in redox ratio was statistically significant (P<0.05) except at 90 min.

Total glutathione deviated statistically significant at all time points except 45 min, GSH only at 90 min and 24 hours, and GSSG deviated at 90 min and 72 hours. No difference was found in the redox ratio of lenses in the two media.

No measurable quantities of either GSH or GSSG could be recovered in the castor oil medium.

### High resolution respirometry

Although addition of antimycin A (an inhibitor of the electron transfer system) caused the respiration rate to nearly cease in all lens samples, from 8.8+/−3.0 to 0.5+/−0.9 pmol/(s*ml) (p<0.01, paired t-test, n = 8), confirming that oxygen consumption was due to mitochondrial activity even after 1 hour of storage in media. No statistical difference could, however, be found between the three experimental groups (removed immediately, stored 1 hour in Optisol-GS and stored 1 hour in castor oil).

### Rat lenses removed 6 hours post mortem

**Figure 2:**
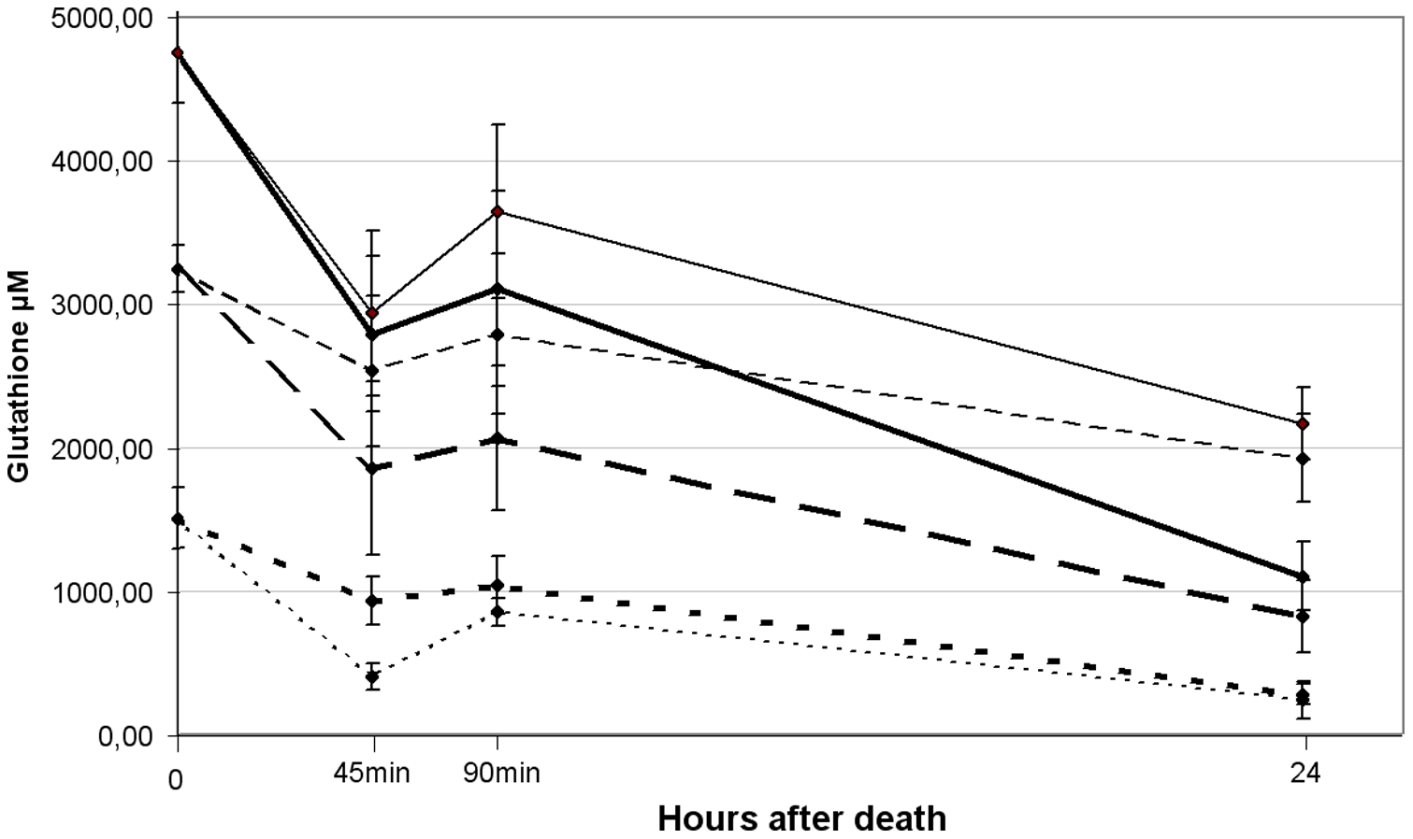
Glutathione in *post mortem* stored lenses. Storage inside the eye retain glutathione in the lens, although affected by a redox shift of decreased GSH and increased GSSG concentrations. GSSG quickly drop to similar levels as *in vitro* stored lenses, whereas GSH is lost at a slower rate without any rapid drops. Total glutathione is shown as full lines, GSH as large dotted and GSSG as small dotted lines. Bold lines show the progression in Optisol-GS and thin lines in castor oil.

The effect of the intact eye environment on the lens was studied by taping the eyes shut after death and storing the animals for 6 hours at 4°C. The initial total glutathione value was 4.76±0.35 mM, an amount very similar to lenses removed after death. GSH values were reduced to 3.25±0.18 mM and GSSG values at an increased amount of 1.51±0.21 mM compared to fresh lenses, giving an initial redox ratio was 2.17±0.18 (Fig 2).

Post mortem lenses stored in Optisol-GS caused total glutathione and GSH to decrease steadily towards 24 hours, with significant deviation from initial values at all time points. Due to the increased initial GSSG concentration it followed a steeper drop towards similar concentrations after 12 hours.

Redox ratio remained constant with only slight variations at 24 hours, and was not found statistically significant at any time points.

Post mortem lenses stored in castor oil decreased in total glutathione steadily throughout the 24 hours, with significant deviation from the initial value at all time points. GSH equally followed a steady decline, although only the concentration at 24 hours deviated significantly from the starting value. As with Optisol-GS media the GSSG concentration dropped towards the constant value found at lenses removed immediately after death, and thus deviated significantly at all time points from the starting value.

A comparison of the two media revealed that the concentration for total glutathione only deviated significantly at 24 hours, whereas GSH concentrations deviated at all time points and GSSG only at 45 min. The redox ratio deviated significantly between the two media except at 90 min.

## Discussion

From our studies, it became clear that lenses stored in castor oil maintained higher levels of glutathione than lenses stored in Optisol-GS. Lenses left within the intact eye 6 hours post mortem showed no loss of glutathione (Fig 2), but levels dropped again after subsequent storage in media. Differences in the rate of GSH loss were most likely due to the availability of oxygen, which supports mitochondrial activity. The data also support that the loss of glutathione in general is due to mechanisms of efflux and degradation which were still functional *in vitro*.

### Glutathione efflux

The lens exhibits a wide range of transport mechanisms for glutathione, mostly in the form of passive transport over the membrane of lens fibres but also active transport in and out of the lens itself over the epithelial barrier. The passage of GSH over the rat lens capsule is facilitated by two transport proteins, Rat Canalicular GSH Transporter (RcGshT) and Rat Sinosoidal GSH Transporter (RsGshT) [17], [18]. These transporters function in a bidirectional manner, transporting GSH along the concentration gradient. In addition, a third transporter, which functions against concentration gradients, has been characterized in rat epithelium [19]. It has been suggested that GSSG can leave the lens by simple diffusion [20]. In this study, we found that increased glutathione concentrations of the media resulted in a statistically significant increase of glutathione levels in *in vitro* Optisol-GS stored lenses, confirming that diffusion of glutathione over the lens epithelium is concentration dependent. Finally, studies on bovine lenses have shown GSH passively traversing the lens capsule in both directions, driven by differences in concentration of glutathione and glucose [21].

In this study, lenses stored inside the eye for 6 hours post mortem retained all of their glutathione (Fig 2) when compared to lenses analyzed immediately after death. The balance of glutathione concentrations in the surrounding humors, established under normal conditions, most likely prevents this loss from diffusing. When these lenses were subsequently transferred to storage media, surrounding glutathione concentrations were lower and passive transport was evidenced by the loss of total glutathione. GSSG levels did not decrease differently in the two media, but rather showed a rapid efflux in both and, after 24 hours, lenses had equal concentrations under these two conditions (Fig 2). Lens GSH loss, however, was much slower in castor oil than Optisol-GS media, a difference most likely due to its lipophobic nature.

In contrast to the lenses removed 6 hours post mortem, *in vitro* lenses were still metabolically active when placed in storage media. High resolution respirometry showed that even after 1 hour in media, these lenses had functioning mitochondria. Mitochondrial activity requires glucose and oxygen, which are only available in Optisol-GS. GSH is readily transported into mitochondria and is essential for their function [22]. This factor would account for the rapid drop of total glutathione and GSH observed in Optisol-GS stored lenses. In addition, sustaining metabolic activities in these lenses would cause an oxidative shift in the intracellular redox state, causing GSH conversion to GSSG. As was seen in post mortem experiments, GSSG readily passes into medium and this factor may also contribute to the rapid loss of glutathione in Optisol-GS (Fig 1). Conversely, a lack of oxygen and nutrients represses metabolism, and GSH levels remained high in castor oil stored lenses during the early time-points analyzed. The slower passive loss that was seen in the post mortem experiments, however, eventually results in the same depletion of glutathione in these lenses after 24 hours.

### Glutathione degradation

In lenses stored in both media the decrease of GSH was not matched by a proportional rise in GSSG and instead an overall loss of glutathione was observed. Glutathione recovery in Optisol-GS media after lens incubation only reached a limit of 30 nmol, a value lower than the 130 nmol lost by the lenses. No glutathione was found in castor oil after storage, most likely due to its non-polar structure which would resist the dissolution of polar GSH molecules. Lenses stored in this media, however, also showed a loss of total glutathione.

These data support the concept that while glutathione can be lost by passive diffusion, it may also be lost by degradation [23], [24]. As glutathione passes out of the lens, γ-glutamyl transferase catalyzes cleavage of the pseudo peptide bond between glutamic acid and cysteine in a non-ATP dependent manner. The γ-glutamyl cycle is integral in the process of recycling glutathione in the lens [25]. Once cleaved, however, the glutathione constituents will no longer be detectable by the assay used here. Normally, these peptides would then re-enter the lens and be used to form new GSH molecules. In media, however, these amino acids are diluted and instead an overall loss of glutathione was observed.

Oxygen saturation of porcine lenses has been shown to take approximately 2 hours [26]. Although the rate at which oxygen reaches the nucleus may differ in the smaller and more compact rat lens, such a delay could explain why the rate at which GSH is lost is not constant but rather increases up until 90 minutes (Fig 1) in the Optisol-GS stored lenses.

### Conclusion

In summary, glutathione measurements provide valuable insight into which storage methods best preserve lenses in their *in vivo* state. This issue is important for studies that require an intact lens, for example morphological or functional evaluations of human donor lenses.

The final amounts of both total and reduced glutathione in castor oil stored lenses were 3 times higher than in Optisol-GS stored lenses after 72 hours. In addition, it was determined that prior to storage in castor oil, lenses are best left within the eye during the early hours after death, in order to maintain *in vivo* levels of glutathione. Storage times of rat lenses remain limited to 24 hours, after which glutathione concentrations reach levels too low for proper representation and reflect an overall deadline for transportation time of stored lenses.
